# Epithelial-Mesenchymal Transition Promotes the Differentiation Potential of *Xenopus tropicalis* Immature Sertoli Cells

**DOI:** 10.1155/2019/8387478

**Published:** 2019-05-05

**Authors:** Thi Minh Xuan Nguyen, Marketa Vegrichtova, Tereza Tlapakova, Magdalena Krulova, Vladimir Krylov

**Affiliations:** Department of Cell Biology, Charles University, Faculty of Science, Vinicna 7, Prague 2 128 44, Czech Republic

## Abstract

Epithelial-mesenchymal transition (EMT) is a fundamental process in embryonic development by which sessile epithelial cells are converted into migratory mesenchymal cells. Our laboratory has been successful in the establishment of *Xenopus tropicalis* immature Sertoli cells (XtiSCs) with the restricted differentiation potential. The aim of this study is the determination of factors responsible for EMT activation in XtiSCs and stemness window acquisition where cells possess the broadest differentiation potential. For this purpose, we tested three potent EMT inducers—GSK-3 inhibitor (CHIR99021), FGF2, and/or TGF-*β*1 ligand. XtiSCs underwent full EMT after 3-day treatment with CHIR99021 and partial EMT with FGF2 but not with TGF-*β*1. The morphological change of CHIR-treated XtiSCs to the typical spindle-like cell shape was associated with the upregulation of mesenchymal markers and the downregulation of epithelial markers. Moreover, only CHIR-treated XtiSCs were able to differentiate into chondrocytes *in vitro* and cardiomyocytes *in vivo*. Interestingly, EMT-shifted cells could migrate towards cancer cells (HeLa) *in vitro* and to the injury site *in vivo*. The results provide a better understanding of signaling pathways underlying the generation of testis-derived stem cells.

## 1. Introduction

Epithelial-mesenchymal transition (EMT) and its reverse process, mesenchymal-epithelial transition (MET), occur in numerous developmental processes including mesoderm, neural crest, and testicular formations [1]. EMT is characterized by the loss of the apical-basal polarity and cell-cell contacts and the increase of cell interactions with the extracellular matrix (fibronectin, vitronectin) [2, 3]. During development, EMT ensures both, high migratory capacity and the cell stemness. Later, EMT-shifted cells can undergo the reciprocal MET to form tissues and organs such as somites [4], kidney [5], and liver [6]. EMT has also been considered to contribute to tissue repair, fibrosis tissue, and cancer metastasis [1].

Recently, several groups have tried to generate mesenchymal stem cells (MSCs) by inducing EMT in cultured human epithelial cells. Indeed, the immortalized human mammary epithelial cells were incubated with recombinant TGF-*β*1 or transduced with vectors expressing EMT key transcription factors, Snai1 or twist. Treated cells have exhibited the characteristics of MSCs, including specific antigenic profile, the capacity to differentiate into mesodermal cell types and to migrate towards cancer cells and wound injury sites [7, 8].

Sertoli cells (SCs) in seminiferous tubules play an essential role in male reproduction. In the early development, the anti-Müllerian hormone (AMH) is secreted by SCs to regress the Müllerian tract contributing to male sex determination and testis formation [9]. In the adult testis, SCs regulate sperm cell production via nutritional and scaffolding supports.

In spite of their importance, the origin of Sertoli cells is still a question. The insertion of the EGFP (enhanced green fluorescent protein) gene under the promoter of SRY (sex-determining region Y), a Sertoli cell-specific gene, or Myc-tag upstream the SRY stop codon has visualized mesenchymal morphology of SCs [10, 11]. Furthermore, cells labelled with 5′-bromo-2′-deoxyuridine (BrdU) beneath the coelomic epithelium migrating into XY and XX/Sry gonads had positive staining with SF1 (steroidogenic factor 1), another Sertoli cell-specific marker [12, 13]. The SC origin in coelomic epithelium has been further suggested in [10, 12, 14]. Taken together, these results indicate the mesenchymal phenotype of pre-Sertoli cells. Their maturation in seminiferous tubules is coupled with a mesenchymal-epithelial transition (MET). Therefore, we assume that the reverse process, EMT, could convert immature Sertoli cells back to the stem cell-like state with expected broader differentiation potential.

Our laboratory has been successful in establishing a cell line of *Xenopus tropicalis* immature Sertoli cells (XtiSCs) in a long-term culture [15]. Germ cell markers were not detected in XtiSCs which confirms their somatic origin. Immunocytochemical staining against Sox9 (SC marker [16]) showed its presence in approx. 90% of the cells. On the other hand, XtiSCs formed compact colonies expressing both vimentin and cytokeratin, the mesenchymal and epithelial intermediate filaments, respectively. These results indicate that we are dealing with immature Sertoli cells [17, 18]. XtiSC allotransplantation into *X. tropicalis* tadpoles revealed their accumulation in many tissues and organs encompassing the heart, intestine, and pronephros. However, immunohistochemistry of tadpole sections showed only the presence of vimentin in transplanted cells but no expression of tissue- or organ-specific markers [15]. XtiSC *in vitro* differentiation potential was also limited (unpublished results).

TGF-*β*1, FGF2, and GSK-3 inhibitor are common EMT inducers of epithelial cells *in vitro* [19–21]. We have employed these factors individually to reverse XtiSC maturation and broaden their differentiation potential. Subsequent evaluation of cell morphology and changes in a gene expression profile after the treatment have been done by reverse transcription polymerase chain reaction (RT-PCR), immunostaining, and flow cytometry. Our results showed that XtiSCs underwent full EMT by pharmacological inhibition with GSK-3 (CHIR99021) and partial EMT using FGF2.

## 2. Materials and Methods

All chemicals were supplied by Sigma unless otherwise stated.

### 2.1. XtiSC Culture and Fluorescent Immunostaining

*Xenopus tropicalis* immature Sertoli cells (XtiSCs) were obtained and cultured as described [15]. To induce epithelial-mesenchymal transition (EMT), cells were cultured in a growth medium overnight before its replacement by induction medium supplemented with CHIR99021 (CHIR; GSK-3 inhibitor, 3 *μ*M, Sigma), mFGF2 (25 ng/ml), or hTGF-*β*1 (2.5, 5, or 10 ng/ml) (all from PeproTech) for 3-4 days. CHIR99021, FGF2, and TGF-*β*1 solutions were prepared according to the manufacturer's instructions. CHIR was dissolved in DMSO at 3 mM stock solution; therefore, the same amount of DMSO (0.1%) was added in the growth medium of control cells. All stock solutions were kept at -20°C until needed. All cell media contained 10% fetal bovine serum (FBS), except the medium supplemented with TGF-*β*1 (only 0.5% FBS).

For immunofluorescence, cells were plated on coverslip glasses coated with collagen type I (2.5 *μ*g/cm^2^) or poly-L-lysine (4 *μ*g/cm^2^) and cultured in the indicated medium for 3-4 days. Cells were then fixed with 2% formaldehyde, permeabilized, and incubated with primary antibodies (list and dilution in Supplementary Table 2) followed by secondary antibodies conjugated with Alexa Fluor-488 or Alexa Fluor-594 (1 : 500, ThermoFisher Scientific). Regarding F-actin staining, fixed cells were incubated with Alexa Fluor-568-conjugated phalloidin (1 : 100, ThermoFisher Scientific) for 1 hour at room temperature (RT). Cell nuclei were visualized by DAPI.

### 2.2. RNA Isolation and RT-PCR

Total RNA was isolated from XtiSCs by the RNeasy Plus Mini Kit (Qiagen) followed by the preparation of cDNA using the RevertAid First Strand cDNA Synthesis Kit (ThermoFisher, Scientific). This process included the on-column DNase treatment step. Target cDNA levels were analyzed by semiquantitative RT-PCR in quadruplicates. Sequences of RT-PCR primers are presented in Supplementary Table 1. The relative gene expression was normalized to an endogenous reference gene *actb* (*β*-actin). The ImageJ program was used to measure the intensity of PCR product on figures of electrophoresis gels.

### 2.3. *In Vitro* Differentiation

The micromass culture technique as described by [22] was employed to differentiate XtiSCs to chondrocytes using differentiation medium from the StemPro™ Chondrogenesis Differentiation Kit (ThermoFisher Scientific) diluted 2 : 1 with water. Cells were cultured in a growth medium as a control. After 10 days, the pellets were fixed and embedded in OCT for cryostat sectioning. Alcian blue staining was used to assess the formation of the extracellular matrix, a hallmark of chondrogenic differentiation. The expression of a chondrogenic marker (collagen type II) was also analyzed by immunofluorescent staining.

For osteogenic differentiation, a medium from the StemPro™ Osteogenesis Differentiation Kit (ThermoFisher Scientific) diluted 2 : 1 with water was used. Only half of the medium was changed every 3-4 days. Control cells were cultured in a standard growth medium. After 21 days, the cells were stained with alizarin red. Quantitation of alizarin red staining was done by the Osteogenesis Quantitation Kit (Millipore).

Adipogenic differentiation of XtiSCs was performed by adding 1 *μ*M dexamethasone, 0.2 mM indomethacin, 0.1 mg/ml insulin, 1 mM 3-isobutyl-1-methylxanthine in LB15, and RPMI medium with 10% FBS to a confluent cell culture. The medium was changed every 4 days. Control cells were cultured in a standard growth medium. After 21 days, adipogenic differentiation was assessed by staining with oil red O.

### 2.4. X-gal Staining

Cells were fixed with 4% formaldehyde for 5 min at RT and then incubated with 0.1% X-gal (Fermentas) in staining solution (5 mM potassium ferrocyanide (K_4_Fe(CN)_6_), 5 mM potassium ferricyanide (K_3_Fe(CN)_6_), 150 mM NaCl, 2 mM MgCl_2_, and 40 mM citric acid in sodium phosphate solution, pH 6.0) at 37°C for 12 hours. Stained (senescent) cells with a blue color were observed and imaged under an inverted microscope (Olympus).

### 2.5. *In Vitro* Migration Assay

**Figure 1:**
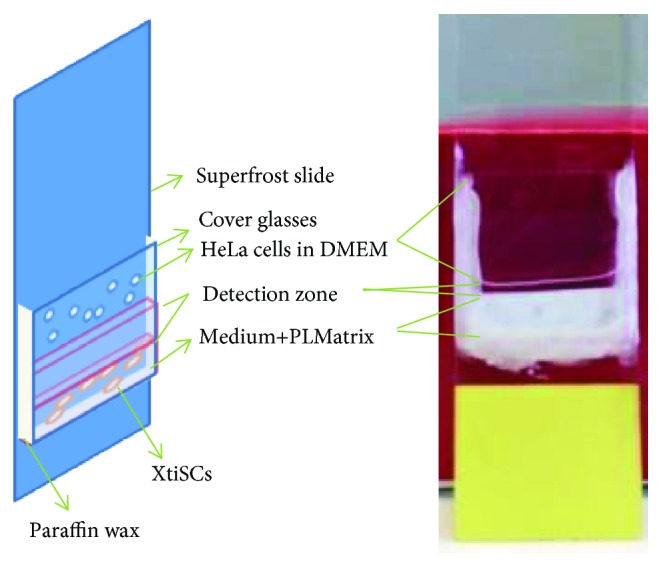
Scheme of the incubation chamber for the directed migration of XtiSCs towards cancer HeLa cells. XtiSCs were placed into lower part of the incubation chamber containing 100 *μ*l of FGF2-, CHIR99021-, or 0.1% DMSO-supplemented media. After three-day treatment, inducing or control (0.1% DMSO) medium was replaced by 120 *μ*l of standard cultivation medium supplemented with PLMatrix. HeLa or HEK cell suspension (control) was added to the upper part after the solidifying of PLMatrix. HeLa cells are separated from XtiSCs by the detection zone.

Directed migration ability of induced XtiSCs towards cancer cells was investigated. Paraffin wax was used to fix a collagen-coated coverslip glass on a superfrost plus slide (ThermoFisher Scientific). The space between the glass and the slide was filled with 100 *μ*l suspension of 1.3 × 10^5^ cells. This chamber was cultured vertically using the indicated medium until reaching confluency (3-4 days). The induction medium was then replaced with 120 *μ*l of standard growth medium (with 0.5% FBS) plus 10% PLMatrix (PL BioScience, Aachen, Germany) to make a detection zone above the cell layer (Figure 1). The backside of the cell growth area was covered with a white adhesive label to distinguish it from the detection zone. HeLa cell suspension was then added after the complete solidification of PLMatrix (about 1 hour in an incubator). HEK 293 cell suspension was used as a control. After 2 days in the matrix, the number of cells migrating into the detection zone was counted based on at least five microscope figures per experimental group. The average of the cell number per mm^2^ is depicted in the chart (Figure 1).

### 2.6. Cell Transplantation and Tadpole Cultivation

*X. tropicalis* embryos were produced and cultivated by the standard *in vitro* fertilization procedure [23]. Transgenic Katushka red fluorescent protein- (RFP-) positive cells were prepared and sorted as described in [15]. Each tadpole was injected with 1000 RFP-expressing cells into the peritoneal cavity using the protocol of [15]. After transplantation, the distribution of RFP-positive cells was observed under a fluorescence stereomicroscope (Olympus). All experiments with tadpoles were performed following institutional-approved protocols.

### 2.7. *In Vivo* Wounding Assay

To analyze the wound homing capacity of XtiSCs, the wounding assay was performed as described [24] with modifications. Briefly, stage 51 or elder (around 3-week-old) *X. tropicalis* larvae were anesthetized with 0.01% tricaine (MS-222) and put into a Petri dish with 6% Ficoll, 0.1x MMR, and 0.1% BSA. Two hundred RFP-positive XtiSCs (40 nl) treated or untreated with CHIR99021 had been microinjected into larvae through dorsal blood vessels near the abdomen. Just after microinjection, the distal third of the tail was wounded by #55 Forceps (Fine Science Tool). Transplanted *X. tropicalis* larvae without wounding were used as a control. The tadpoles were imaged after 6 hours under the fluorescence stereomicroscope (Olympus). Two days later, the wounded tadpoles were collected, fixed, and sectioned for immunohistochemistry employing the antibody against fibronectin.

### 2.8. Immunohistochemistry

Testes or transplanted tadpoles at indicated time points were collected, fixed overnight in MEMFA (0.1 M MOPS, 2 mM EGTA, 1 mM MgSO_4_, and 3.7% formaldehyde) at 4°C, rinsed, and immersed into 1.8-3% agarose depending on the sample rigidness. Vibratome sectioning and immunohistochemistry of fixed tadpoles were performed as described in [15]. 30-35 *μ*m thick sections showed good morphology and were suitable for immunostaining. For double staining, primary antibodies were chosen from different host animals (mouse and rabbit). To eliminate the background by crossing excitation and emission wavelengths between fluorescent dyes, secondary antibodies conjugated with two fluorophores at different spectra (Alexa Fluor-488 and Alexa Fluor-594) were employed. Individual sections were mounted on slides with Mowiol/DAPI mounting medium and observed under a fluorescent microscope (Olympus Cell-R). Z-stack images for every single layer have been acquired and presented to avoid mixing fluorescent signals in different optical layers.

### 2.9. Dissociation of Tadpoles and Flow Cytometry Analysis

Injected tadpoles at the indicated time points were collected, anesthetized with 0.4% tricaine (Sigma-Aldrich), and immersed into 0.05% collagenase type 1A for 1 hour and then into 0.025% trypsin-EDTA at 37°C for 20-30 minutes. Tadpoles were pipetted several times until completely dissociated and filtered through 20 *μ*m cellTrics. Cells were collected by centrifugation and suspended in PBS for flow cytometry analysis.

Cells were washed with PBS, collected by scrapers, fixed with 4% formaldehyde for 10 minutes, rinsed, and incubated with primary antibody against CD29 (DSHB 8C8, 2 *μ*g/ml) for one hour at RT, following staining with the Alexa Fluor-488-conjugated secondary antibody (ThermoFisher Scientific, 1 : 500) for 30 minutes. Cells incubated only with secondary antibody were used as a control. Stained cells or dissociated tadpoles were analyzed by a flow cytometer (BD LSR II) accompanied with the BD FACSDIVA™ software for data analysis.

### 2.10. Statistical Analysis

All assays were repeated in at least three independent experiments; the exact number of experiments were mentioned in each figure. All data were expressed as mean ± standard deviation (SD). For evaluation of group differences, unpaired Student's *t*-test was used assuming equal variance with data from three independent experiments and three repetition with each experiment. A *p* value of < 0.05 was accepted as significant.

## 3. Results

### 3.1. Cytokeratin as a Marker of Immature Sertoli Cells in *X. tropicalis* Testis

**Figure 2:**
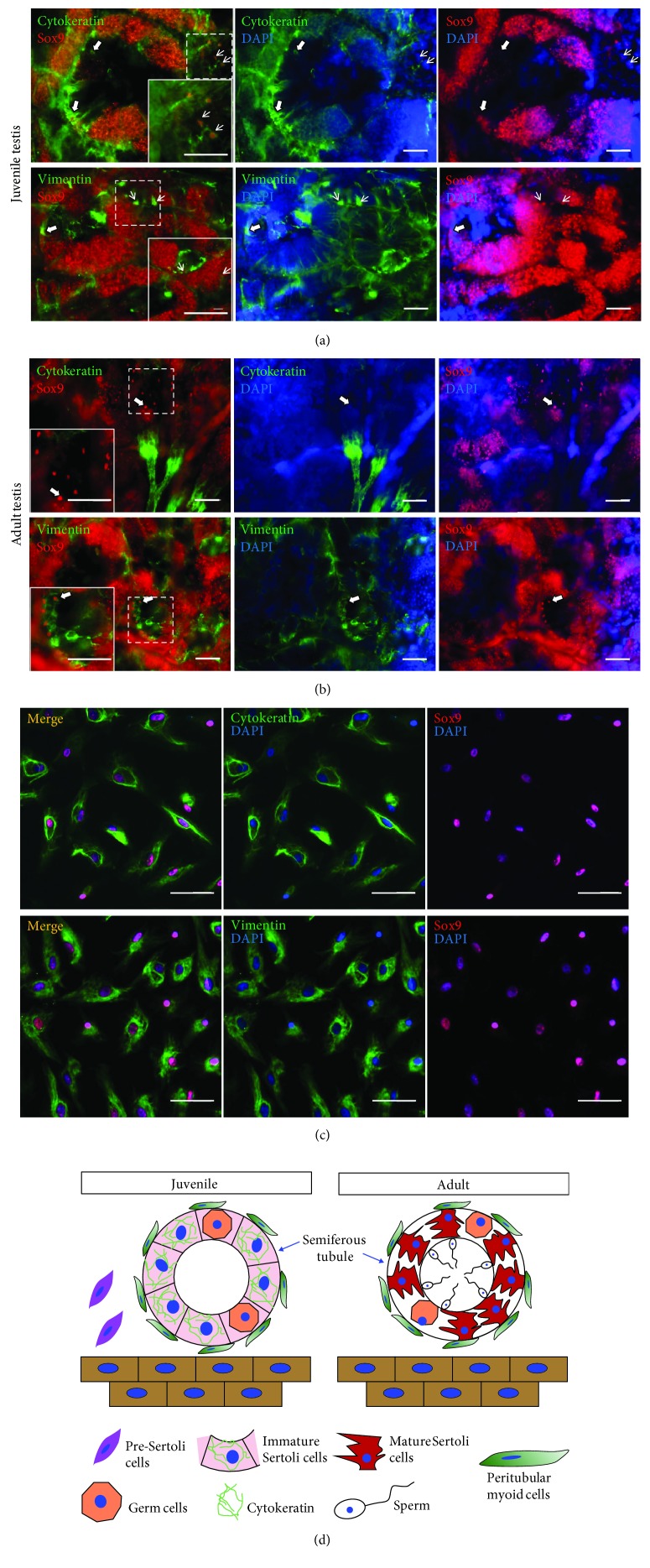
(a) Testicular cross-sections from juvenile *Xenopus tropicalis* stained with two antibodies, cytokeratin (green)/Sox9 (red, SC marker) and vimentin (green)/Sox9. Thin arrows show migrating Sertoli cells (SCs) or pre-SCs outside the seminiferous tubules. Thick arrows indicate SCs inside the tubules. Cytokeratin is absent in pre-SCs, in contrast to tubular SCs. On the other hand, vimentin is present in both types, migrating and tubular SCs. (b) Double staining of testicular cross-sections with two antibodies, cytokeratin (green)/Sox9 (red) and vimentin (green)/Sox9 from adult *Xenopus tropicalis*. The only vimentin is observed in Sox9 nuclear-expressing SCs. (c) Immunofluorescent staining of XtiSCs with antibodies against cytokeratin (green)/Sox9 (red) shows that most of the XtiSCs expressed both proteins which evidence them as immature Sertoli cells or SC progenitors. The expression of vimentin was observed in XtiSCs as well. Nuclei were stained with DAPI. Scale bar: 50 *μ*m. (d) The scheme illustrates the development of Sertoli cells in testis.

Changing the expression of various intermediate filaments in Sertoli cells has been reported elsewhere [17, 18]. To test the expression of two IFs, cytokeratin and vimentin, in *X. tropicalis* SCs, testicular sections from juvenile (6-month-old) and adult (3-year-old) *X. tropicalis* were employed for double staining with two antibodies against cytokeratin/Sox9 or vimentin/Sox9 (Sox9, a specific marker of SCs). In the juvenile testes, SCs with the Sox9 accumulation in the nuclei were observed both inside seminiferous tubules and outside (migrating SC progenitors) (Figure 2(a)). Regarding adult frogs, most of the SCs were localized inside tubules (Figure 2(b)). In contrast to the stable presence of vimentin, the expression of cytokeratin was very dynamic. There was little to no cytokeratin in migrating and adult Sertoli cells. On the other hand, this intermediate filament was strongly detected in tubular SCs in juvenile testes (Figures 2(a) and 2(b)). These results indicate that cytokeratin accompanied with Sox9 can be used as markers of immature Sertoli cells.

*Xenopus tropicalis* immature Sertoli cells have been isolated and characterized as described in [15]. Double immunofluorescent staining with two antibodies against cytokeratin and Sox9 showed that most of the cells (more than 90%) expressed both proteins indicating their immature state (Figure 2(c)). The expression of vimentin was observed as well (Figure 2(c)).

### 3.2. Pharmacological Inhibition of GSK-3 by CHIR99021 Induces EMT in XtiSCs

**Figure 3:**
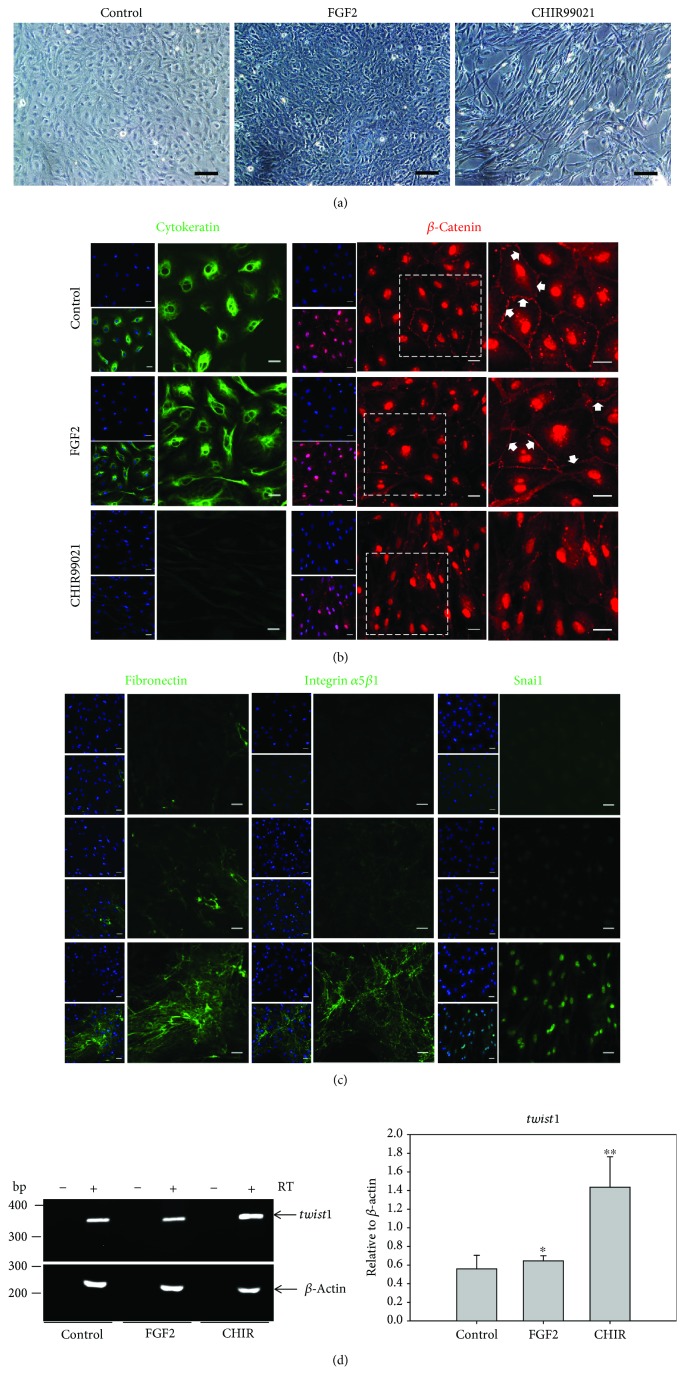
GSK-3 inhibitor (CHIR99021) stimulates EMT in XtiSC cell culture. XtiSCs were cultured in a growth medium supplemented with 25 ng/ml FGF2 or 3 *μ*M CHIR99021 or 0.1% DMSO as a control. (a) After three-day treatment, the morphological change of XtiSCs from cobblestone shape to a long-rod shape in a medium with CHIR99021 was observed. Scale bar: 100 *μ*m. (b, c) Immunofluorescent staining and RT-PCR analysis showed the downregulation of epithelial markers (cytokeratin, *β*-catenin at the plasma cell membrane) (b) and the increase of mesenchymal markers (fibronectin, integrin *α*5*β*1, Snai1, and *twist1*) (c, d). Arrows show the expression of *β*-catenin at the plasma cell membrane. Nuclei stained with DAPI. Scale bar: 20 *μ*m. Results are representative of three biological replicates; ^∗^ *p* < 0.5, ^∗∗^ *p* < 0.001.

**Figure 4:**
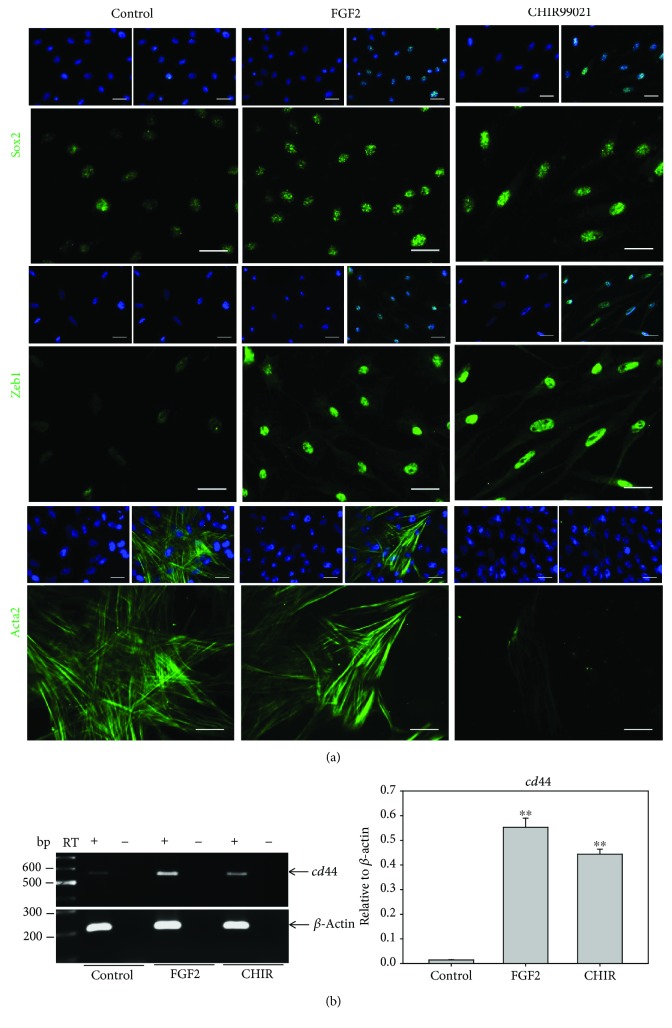
Gene expression of mesenchymal stem cell markers in FGF2- and CHIR99021-treated XtiSCs. Immunofluorescent staining (a) and RT-PCR (b) show the increased expression of stem cell markers, Sox2, *cd44*, and Zeb1 after FGF2 and CHIR99021 treatments. However, only CHIR-treated XtiSCs suppressed Acta2, the earliest marker of mural cell progenitors. RT: reverse transcriptase. Scale bar: 20 *μ*m. Nuclei stained with DAPI. Results are representative of three biological replicates; ^∗∗^ *p* < 0.01.

The expression profile and proliferative activity confirm the SC origin of XtiSCs and their immature phenotype. To answer our hypothesis about reprogramming immature SCs back to their stem cell state by the EMT process, we added GSK-3 inhibitor, FGF2, or TGF-*β*1 into the XtiSC culture. Three-day incubation of XtiSCs with 3 *μ*M CHIR99021 increased the expression of EMT transcription factors such as Snai1, Zeb1 as revealed by immunofluorescent staining (Figures 3(c) and 4(a)), and *twist* by RT-PCR analysis (Figure 3(d)). Spindle-like cell shape (Figure 3(a)) and the downregulation of cytokeratin (Figure 3(b)) were also observed after the treatment with GSK-3 inhibitor. On the other hand, cell-cell junctions were disrupted altogether with the disappearance of *β*-catenin on the cell membrane (Figure 3(b)). Alternatively, cell-matrix adhesions were facilitated through the increase of fibronectin and its receptor, integrin *α*5*β*1, deposited on a collagen-coated surface in the culture medium supplemented with CHIR99021 (Figure 3(c)). Other mesenchymal proteins, including vimentin and CD29, remained unchanged in all groups (Figure S2).

XtiSCs treated with FGF2 upregulated Zeb1 expression (Figure 4(a)) but not other mesenchymal markers nor downregulated epithelial markers, such as cytokeratin (Figures 3 and 4(a)).

TGF-*β*1 was also used to induce EMT. However, after 3-day treatment at the concentration of 2.5 ng/ml or 5 ng/ml, XtiSCs turned into bigger and flat-shape cells (Figure S3A) accompanied by the higher activity of *β*-galactosidase at pH 6.0 (positive staining with X-gal) (Figure S3B). All cell groups also expressed cytokeratin (Figure S3C). However, only TGF-*β*1-treated cells formed the thick and parallel stress fibers as visualized by F-actin staining with phalloidin. These results indicated that XtiSCs became senescent. At the concentration of 10 ng/ml, most of the cells died after 24 hours (data not shown). The same results were observed after treatment with TGF-*β*1 at different time courses (5 days and 7 days) (data not shown). Therefore, results presented in this article include only experiments employing FGF2 and CHIR99021.

Taken together, XtiSCs underwent full EMT after the incubation with GSK-3 inhibitor (CHIR99021) by upregulation of mesenchymal and downregulation of epithelial markers. FGF2 treatment resulted in only partial EMT accompanied with the upregulation of Zeb1.

### 3.3. EMT Promotes the Stemness and Migration Potential of XtiSCs *In Vitro*

In this part, we examined the stemness of EMT-shifted XtiSCs *in vitro*. Interestingly, either full EMT (CHIR99021) or partial EMT (FGF2) led to the upregulation of pluripotent stem cell marker Sox2 and mesenchymal stem cell surface marker *cd44* (Figures 4(a) and 4(b)). Flow cytometry analysis revealed the expression of another mesenchymal stem cell surface marker CD29 in more than 90% of the cells (Figure S2). However, only CHIR-treated XtiSCs suppressed smooth muscle actin (Acta2), the earliest marker of mural cells (Figure 4(a)). This result indicates that the GSK-3 inhibitor has induced XtiSC reprogramming.

**Figure 5:**
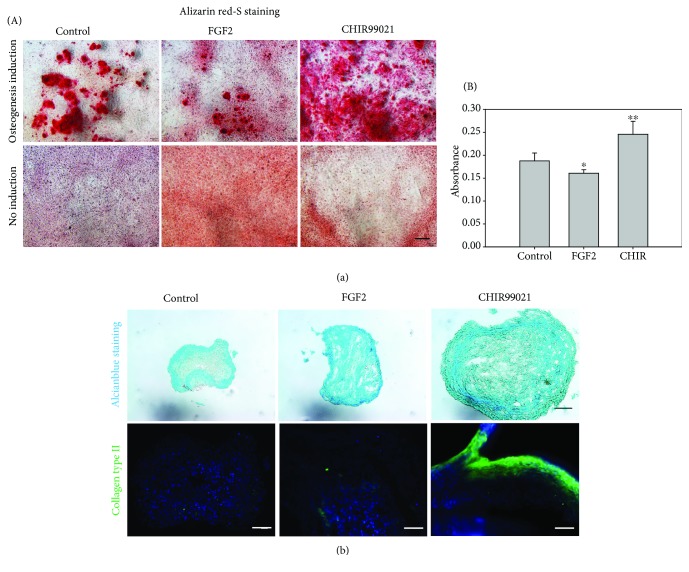
*In vitro* differentiation of XtiSCs. XtiSCs were treated with FGF2, CHIR99021, or 0.1% DMSO as a control for 3-4 days followed by the replacement of medium with osteogenic and chondrogenic induction media. (a) Osteocyte differentiation of XtiSCs was evidenced as a calcium deposit reacting with alizarin red (A). Cells were lysed at low pH to release alizarin red dye from the stained monolayer for a colorimetric test as illustrated in diagram (B). (b) Alcian blue was used to stain glycosaminoglycans in cartilages. In the control (untreated cells), the dye reacts only with the periphery of cell clusters. On the other hand, alcian blue staining is strong and uniform in XtiSC clumps after FGF2 and CHIR99021 treatments, indicating the high level of proteoglycan forming the extracellular matrix. However, collagen type II, a specific marker of the cartilage matrix, was expressed in cells pretreated with CHIR99021 only. DAPI was used as cell counterstains. Results are representative of three biological replicates. Scale bar: 100 *μ*m for bright field images and 50 *μ*m for fluorescent images (lower panel of (b)); ^∗^ *p* < 0.005, ^∗∗^ *p* < 0.001.

Next, *in vitro* differentiation of untreated and treated XtiSCs into three typical mesenchymal cell lineages was investigated. All of the three experimental group cells could differentiate into osteocytes and adipocytes (Figure 5, S4). However, CHIR-treated cells showed higher efficiency of differentiation in comparison to the vehicle or FGF2. In the osteogenic induction medium, pretreatment with CHIR99021 increased the number of alizarin red-positive cells as revealed by colorimetric analysis of the cell lysate (Figures 5(a) and 5(b)). For chondrocytes, cells were induced by the micromass culture technique, and only cells in the induction medium could form pellets. After 2 weeks, cell pellets were collected and sectioned for the alcian blue staining and immunofluorescence analysis using antibody against collagen type II. The size and shape of pellets differed among experimental groups. The biggest and round-shaped spheres were characterized for CHIR-treated cells. Moreover, they also expressed collagen type II, a cartilage specific marker (Figure 5(b)). These data confirmed the mesenchymal origin of XtiSCs and their reprogramming to stem cell-like cells using GSK-3 inhibitor.

**Figure 6:**
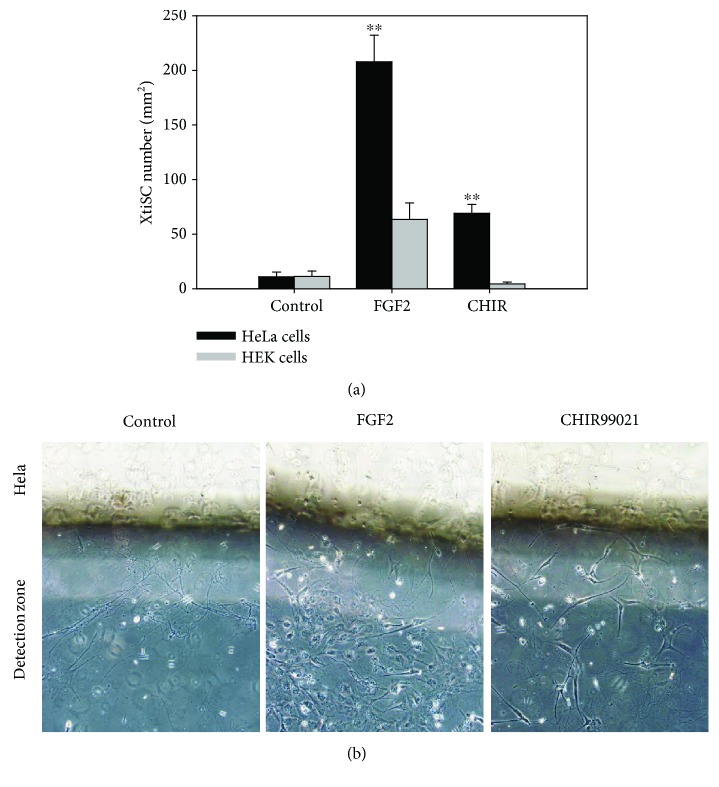
Directed migration of XtiSCs towards cancer HeLa cells. Control XtiSCs or XtiSCs treated with FGF2 or CHIR 99021 were placed into lower part of the incubation chamber as described in Figure 1. After 36-48 hours, the number of cells in the detection zone was counted and expressed in the chart (a). The results are representative of five biological replicates. ^∗∗^ *p* < 0.01. XtiSCs captured in the detection zone are seen in (b).

Furthermore, directed migration towards cancer cells, another prominent feature of mesenchymal stem cells (MSCs) and EMT-shifted cells, was analyzed as well. In this study, we employed human cancer cells (HeLa) as an attractant and HEK cells as a negative control. In order to improve the cell leakage of commercial Boyden chambers, we designed the *in vitro* migration assay in which cells were cultured vertically and migration inducer (HeLa cell suspension) was placed in the upper layer. XtiSCs and the attractant were separated from each other by the “detection zone” based on a semisolid cultivation medium (Figure 1). Both FGF2 and CHIR99021 enhanced the XtiSC migration into the detection zone towards HeLa cells during the 2-day assay. Interestingly, collective migration of FGF2-treated cells increased their number in the detection zone compared with the individually migrated CHIR99021 counterparts (Figures 6(a) and 6(b)).

### 3.4. EMT Promotes the XtiSC Stemness and Migration Potential *In Vivo*

**Figure 7:**
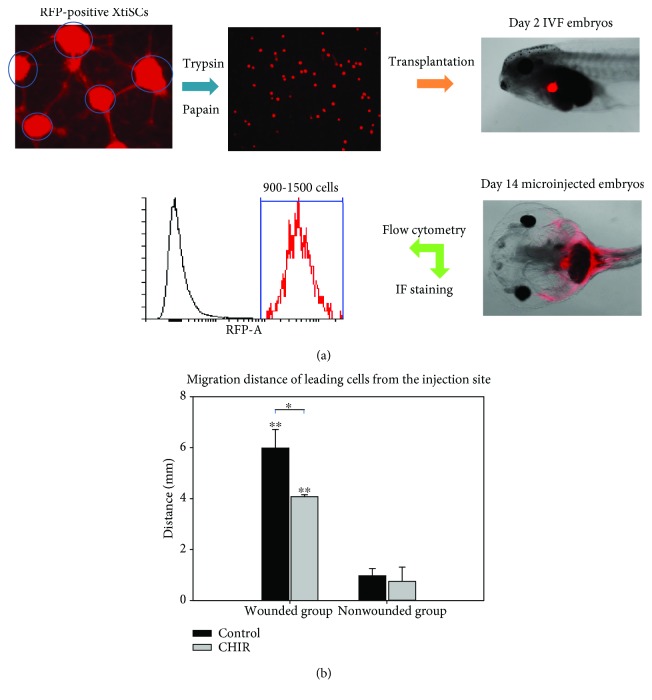
*In vivo* differentiation of EMT-induced XtiSCs into cardiomyocytes. (a) Experimental scheme. RFP-positive XtiSCs were cultured in a growth medium supplemented with 3 *μ*M CHIR99021 or 0.1% DMSO as a control for 3-4 days before transplantation into 2-day-old tadpoles. (b) At 4th, 14th, or 30th day postinjection (dpi), tadpoles were fixed and sectioned for double staining with antibodies against red fluorescent protein and cardiac troponin T labeling cardiomyocytes in the heart. Scale bar: 20 *μ*m. Nuclei stained with DAPI. Results are representative of four biological replicates.

To validate the stemness of EMT-shifted XtiSCs, we have transplanted the transgenic XtiSCs expressing red fluorescent protein (RFP) into *X. tropicalis* embryos and determined their *in vivo* differentiation potential by the expression of specific tissue markers. The similar characteristics of RFP-positive XtiSCs to nontransgenic cells have been revealed in previous study [15]. Approximately 1000 untreated or treated (FGF2 or CHIR99021) RFP-expressing XtiSCs were microinjected into the peritoneal cavity of 2-day-old *X. tropicalis* tadpoles (stage 41) (Figure 7(a)). Two weeks after injection, 900-1500 transplanted cells were detected in each individual using flow cytometry (Figure 7(a)), indicating their high survival rate.

*In vivo* differentiation potential was demonstrated as the ability of XtiSCs to differentiate into cardiomyocytes after allotransplantation. Vibratome sections of tadpoles at 4, 14, and 30 days postinjection (dpi) were stained with rabbit anti-RFP (red) and mouse cardiac troponin T (green) antibodies. Staining with the cardiomyocyte marker prior to microinjection was negative for both treated and untreated XtiSCs (data not shown). 30 days after transplantation, in 30% of the tadpoles (3/10), CHIR99021-treated cells expressed cardiac troponin T inside the heart and were infiltrated in the myocardium (Figure 7(b)). Untreated XtiSCs were distributed around the aorta and heart and were negative for cardiac troponin T (Figure 7(b)). As for the FGF2 experimental group, there was no difference between treated and untreated cells (data not shown). Furthermore, we have examined the markers of other cell types, including chondrocytes (collagen type II), muscle cells (tropomyosin), myofibroblast (Acta2), and neurons (neuronal surface marker, zn12), on transplanted cells as well; however, no positive staining of XtiSCs has been found. In spite of that, CHIR-treated cells have shown some interesting features after their transplantation into tadpoles. They were often found attached to neurons and vascular muscle cells and copied the morphology of these cell types (Figure S5).

**Figure 8:**
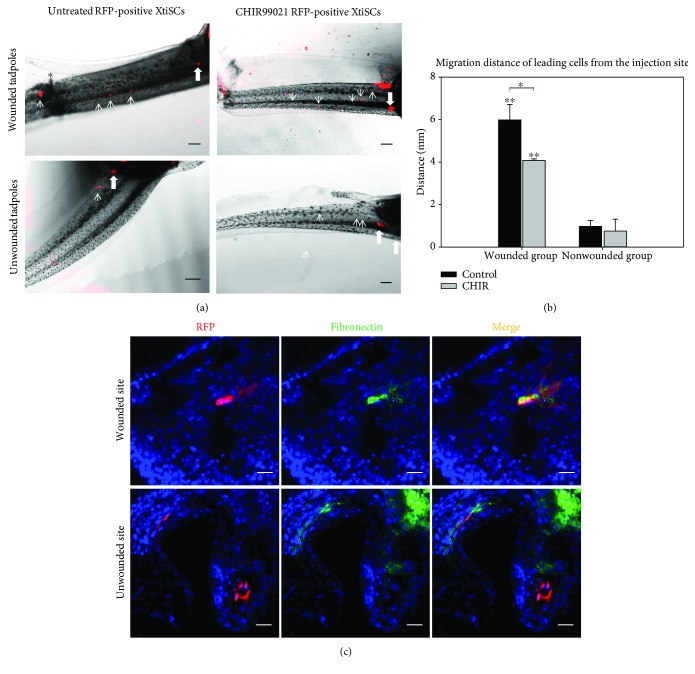
*In vivo* migration of untreated or CHIR99021-treated XtiSCs transplanted into the dorsal veins of 3-week-old tadpoles towards skin injury. (a) Differential migration behavior of untreated and treated RFP-expressing XtiSCs inside wounded or unwounded tadpoles. Arrows show the migrating RFP-positive XtiSCs; thick arrows indicate the injection sites. The injury site is marked by a black asterisk. Scale bar: 500 *μ*m. (b) The distance of leading cells from the injection site was measured 6 hours after transplantation by ImageJ and expressed in the chart. (c) Two days after transplantation, fibronectin was detected in untreated XtiSCs at the injury site only but not in cells outside wounded areas in both groups. Results are representative of three biological replicates. Nuclei were stained with DAPI. Scale bar: 20 *μ*m; ^∗^ *p* < 0.05, ^∗∗^ *p* < 0.01.

We also evaluated the migration potential of XtiSCs transplanted into 3-week-old tadpoles with a cutaneous injury. The distal third of the tail was wounded at 6 mm distance from the injection site using fine forceps. Nonwounded tadpoles with transplanted cells were used as a control (Figure 8(a)). RFP-expressing XtiSCs migrated towards the injury site and accumulated here 6 hours after the transplantation. In nonwounded tadpoles, XtiSCs were found only in the injection sites during the same time period (Figure 8(a)). Unexpectedly, untreated XtiSCs migrated significantly faster towards the injury sites than CHIR99021-treated cells (Figures 8(a) and 8(b)). We also examined the expression of fibronectin 2 days postinjury. Only XtiSCs aggregated at the injury site were positive but not cells migrated elsewhere (Figure 8(c)). This means that XtiSCs have responded to injury signals and contributed to wound healing by expressing fibronectin, an essential component of the extracellular matrix to recover the epidermal layer.

## 4. Discussion

The change in the expression of the intermediate filament, cytokeratin, during Sertoli cell (SC) maturation has been revealed by immunohistochemistry and immunoblotting in the human and mouse. Cytokeratin is detected in the basal part of fetal SCs around the well-differentiated seminiferous cords up to the early postnatal period. However, it is not present in pre-SCs or mature SCs in the adulthood [25, 26]. On the contrary, vimentin staining is intense and surrounds nuclei of mature SCs [17]. Even though these changes are poorly understood, coexpression of cytokeratin and vimentin has been considered as the marker of immature SCs [18]. The same results have been obtained on *Xenopus tropicalis* testicular sections using double immunofluorescent staining with two antibodies against cytokeratin and Sox9 or vimentin and Sox9 (Figures 2(a) and 2(b)). These findings suggest similar mechanisms of Xenopus and human testicular developments. Moreover, the same analysis illustrated the incomplete differentiation state of XtiSCs towards SCs via the expression of all three markers (Sox9, cytokeratin, and vimentin) (Figure 2(c)).

**Figure 9:**
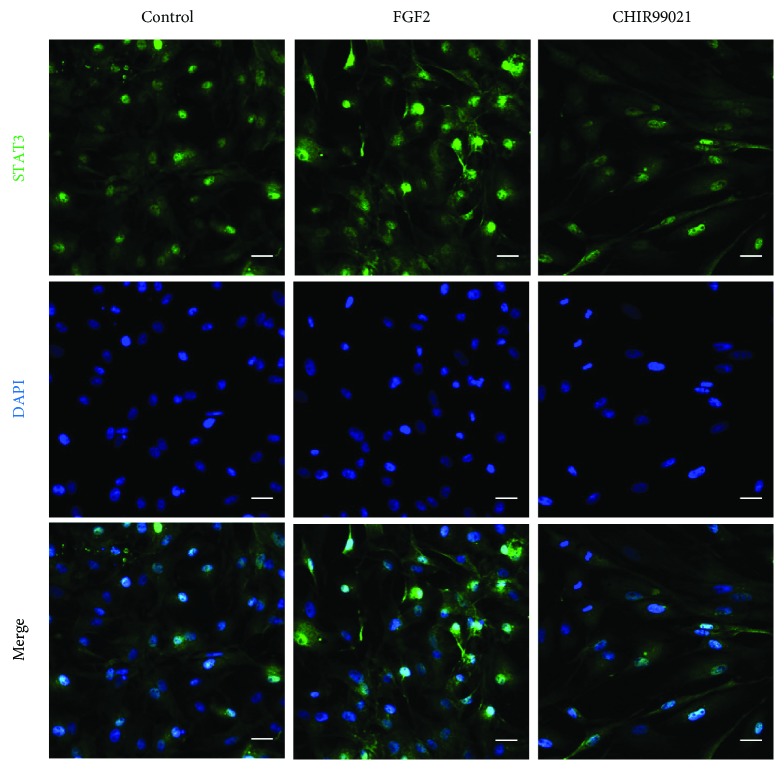
The representative fluorescent images of STAT3 expression in XtiSCs in different medium cultures of three biological replicates. The STAT3 upregulation is observed in FGF2-treated cells. On the other hand, CHIR99021 is responsible for its downregulation. Results are representative of three biological replicates. Nuclei were stained with DAPI. Scale bar: 20 *μ*m.

Signal transducer and activator of transcription 3 (STAT3) plays a dual role during EMT stimulation. Its overexpression enhanced invasion and metastasis of colorectal carcinoma by the downregulation of E-cadherin and by increasing N-cadherin and vimentin expression [27]. However, Lee et al. showed that STAT3 promoted proteasomal degradation of Snai1 through the activation of GSK-3*β*-mediated phosphorylation leading to its ubiquitination in colorectal cancer cells and subsequent EMT suppression [28]. Moreover, GSK-3*β* also regulates the activation of STAT3. STAT3 DNA-binding activity was blocked in GSK-3*β* knockdown or/and GSK-3*β* inhibitor-treated mouse primary astrocytes and microglial cells [29]. Taken together, STAT3 differentially regulates an EMT regarding its epithelial or mesenchymal shift. STAT3 is required to initiate EMT; however, its inhibition is necessary for an EMT completion. Our results are in agreement with the research of [28, 29]. Previous study reported that XtiSCs expressed mesenchymal markers including vimentin and alpha-smooth muscle actin and possessed migration capacity after their transplantation into *X. tropicalis* tadpoles [15]. In spite of that, they still expressed epithelial markers, such as cytokeratin and *β*-catenin, at the plasma membrane (cell-cell adhesions) (Figure 3(b)). These results indicate that XtiSCs attain a hybrid epithelial/mesenchymal state. XtiSC treatment with GSK-3 inhibitor (CHIR99021) reduced STAT3 activity (Figure 9) and upregulated Snai1 expression (Figure 3(c)) to drive XtiSCs to a full mesenchymal state. On the other hand, FGF2 upregulated STAT3 (Figure 8) in XtiSCs; hence, these cells still kept their hybrid phenotype. Consequently, the broader differentiation potential of EMT-shifted XtiSCs was evidenced by the higher efficiency in osteogenic and chondrogenic inductions *in vitro* and into cardiomyocytes *in vivo*. Furthermore, even though staining of transplanted EMT-shifted XtiSCs with other tissue markers was negative, they were observed closed to neurons and vascular muscle cells and copied the morphology of these cell types (Figure S5). These results may indicate that they tended to differentiate into these cell types, but probably, they were immature cells which do not express the functional proteins tested.

In addition, we found that cells treated with GSK-3 inhibitor could grow only on collagen type 1-coated slides or on the gamma-irradiated plastic surface but not on the noncoated or poly-L-lysine-coated slides (Figure S1). This observation may correlate with the deposition of fibronectin on collagen or on the positively charged plastic surface to form an ECM supporting the attachment of transiting cells lacking the cell-cell contacts. This result is in agreement with human epithelial cells, alveolar type II A549s, and human bronchial cells. The upregulation of collagen type I accompanied with the fibronectin-coated surface induced the production of EMT more effectively than the poly-L-lysine layer [30]. Furthermore, collagen type 1 or fibronectin also promoted the motility of EMT-induced HT29 cells as compared to poly-L-lysine coating [31].

Depending on the signaling, epithelial cells can undergo both, partial EMT where the cells exhibit the mixture of epithelial and mesenchymal phenotypes and full EMT where epithelial markers are completely downregulated. The partial EMT enables cells to migrate collectively (as a cell sheet or chain) and maintain a dynamic equilibrium among two phenotypes. Moreover, the stemness of EMT-shifted cells is not relevant to the full mesenchymal state. Rather, a hybrid phenotype is closer to the “stemness window” as defined by [32]. During the EMT process, a stemness window is close to a pure mesenchymal state or pure epithelial state correlated with the initiating signaling of EMT and cell context as well. Among the EMT transcription factors, ZEB1 is important to acquire stemness of EMT-shifted cells via miRNA regulation such as miR-200 [33, 34]. Despite the thorough investigation of interconnection between different EMT phenotypes and stemness in cancer cells, these researches are quite restricted regarding physiological condition. This may correlate with the limitation of available epithelial cell lines able to undergo EMT *in vitro*. Attractively, XtiSCs represent a suitable model with the ability to exhibit a broad spectrum of EMT phenotypes using various growth factors or inhibitors. The range includes the hybrid (more epithelial) state, partial EMT closed to the mesenchymal state with FGF2 treatment, and the complete EMT with GSK-3 inhibitor. The increase of stem cell markers (Sox2, c*d44*) (Figures 4(a) and 4(b)), the upregulation of integrin *α*5*β*1 (Figure 3(c)) which is absent in SCs [35] but present in mesenchymal stem cells [36], and the suppression of Acta2, an earliest marker of mural cells, evidenced the XtiSCs shifting back to the stem cell state by the pharmacological inhibitor, CHIR99021. All the groups of XtiSCs showed the capacity to differentiate into osteocytes and adipocytes (Figure 5(a), S4) *in vitro*; however, the differentiation potential was broadened into chondrocytes and cardiac myocytes after the cell treatment with CHIR99021 (Figures 5(b) and 6(b)).

Mouse mammary epithelial (NMuMG) and mouse cortical tubule (MCT) cells are only two nontransformed cell lines which can undergo EMT *in vitro* [37]. However, their EMT activation is restricted rather to TGF-*β* than other factors. This compromises studies of EMT stimulation via other signaling pathways in nonneoplastic epithelial cells. Interestingly, XtiSCs have shown similar changes in a gene expression level during EMT compared to mammary cells (Figures 3(a) and 4(a)). Therefore, XtiSCs provide an additional model for the investigation of EMT signaling and the stemness of EMT-shifted cells.

The loss of epithelial characteristics is coupled with both, the breakdown of cell-cell contacts and the upregulation of extracellular matrix adhesions, to increase the migration capacity. Surprisingly, CHIR99021-induced XtiSCs (full EMT phenotype) migrated slower than cells exhibiting the hybrid state (FGF2 treatment) *in vitro*. The *cd44* mRNA level of FGF2-treated XtiSCs increased 56-fold, compared to the control, and 1.27-fold, compared to CHIR-treated cells. The increase of CD44 after FGF2 treatment enhanced the directed migration of several cell types, including periodontal ligament cells [38], endothelial cells, and melanoma cells [39].

EMT has been considered to be associated with the reepithelialization process during wound healing where epithelial sheets of primary keratinocytes are able to migrate to the injury site to form a new epidermal layer [40]. This implies that during the healing process, the epithelial cells undergo partial and reversible EMT as a response to inflammatory cytokines such as tumor necrosis factor alpha (TNF-*α*) [41]. Nevertheless, the effect of TNF-*α* on MSCs in injured tissue is more complex. Firstly, TNF-*α* activates MSCs to secrete the paracrine factors stimulating a homing and angiogenesis of endothelial progenitors [42, 43]. Later on, TNF-*α* promotes MSC migration towards the injury site and induces their differentiation [44, 45]. In our study, the response of untreated XtiSCs (hybrid phenotype) to a skin injury was faster and more effective with the expression of fibronectin than in the case of fully mesenchymal XtiSCs induced by CHIR99021 (Figure 8).

## 5. Conclusions

In conclusion, our results suggest the association of EMT with the formation of testicular stem cells. EMT-shifted XtiSCs have brought similar characteristics of mesenchymal stem cells including the capacity to differentiate to the chondrocytes, osteocytes, and adipocytes and directed migration towards cancer cells *in vitro* and towards the injury site *in vivo*. XtiSCs can become a potential model for studying EMT signaling.

## Data Availability

The data used to support the findings of this study are included within the article.

## Supplementary Materials

Supplementary Materials Supplementary Figure 1: phase-contrast images of XtiSCs on microscopic glass coated with various materials. CHIR99021-treated XtiSCs were able to grow only on the collagen type I-coated glass but not on the noncoated or poly-L-lysine-coated glass. In contrary, cells with 0.1% DMSO (control) were not affected by the coating materials. Scale bar: 100 *μ*m. Supplementary Figure 2: (A) immunofluorescent images of mesenchymal interfilament (vimentin) in XtiSCs untreated or treated with CHIR99021 or FGF2. (B) Flow cytometry analysis of surface marker CD29, typical for the mesenchymal cells. GSK-3 inhibitor has no effect on the expression of both proteins. Scale bar: 20 *μ*m. Supplementary Figure 3: XtiSCs became senescent after 3 days of treatment with TGF-*β*1. Phase-contrast images (A), X-gal (B), and phalloidin (F-actin) staining (C) showed the XtiSC change of shape into bigger and flat cells and the increase of galactosidase activity, a senescent marker after TGF-*β*1 treatment. XtiSCs untreated or treated with TGF-*β*1 still expressed the epithelial intermediate filament, cytokeratin (D). Phase-contrast images with scale bar: 100 *μ*m. Nuclei were stained with DAPI. Immunofluorescent images with scale bar: 20 *μ*m. Supplementary Figure 4: adipocyte differentiation of XtiSCs as evidenced by oil red staining after 14 days in induction medium. Positive cells containing red oil droplets appeared in all the cell groups. However, the efficiency of adipocyte induction was quite low. Scale bar: 100 *μ*m. Supplementary Figure 5: immunofluorescent images of cross-sections of injected tadpoles with CHIR-treated cells. Double staining of antibodies against the neuronal surface marker or tropomyosin (a marker of muscle cells) (mouse, green) and RFP (rabbit, red) was employed on the vibratome sections of transplanted tadpoles after 15 days. Scale bar: 50 *μ*m. DAPI (blue) was used for staining cell nuclei. Supplementary Table 1: primer sequences for RT-PCR of *Xenopus tropicalis* transcripts. Supplementary Table 2: antibodies used in immunofluorescent staining.
