# Erratum to “Epithelial-Mesenchymal Transition Promotes the Differentiation Potential of *Xenopus tropicalis* Immature Sertoli Cells”

**DOI:** 10.1155/2019/4081950

**Published:** 2019-08-06

**Authors:** Thi Minh Xuan Nguyen, Marketa Vegrichtova, Tereza Tlapakova, Magdalena Krulova, Vladimir Krylov

**Affiliations:** Department of Cell Biology, Charles University, Faculty of Science, Vinicna 7, Prague 2 128 44, Czech Republic

In the article titled “Epithelial-Mesenchymal Transition Promotes the Differentiation Potential of *Xenopus tropicalis* Immature Sertoli Cells” [1], due to a production error, the horizontal description of Figure 3(c) was missing. Also, Figure 7(b) was incorrectly replaced by Figure 8(b). The correct figures are as follows:

## Figures and Tables

**Figure 1:**
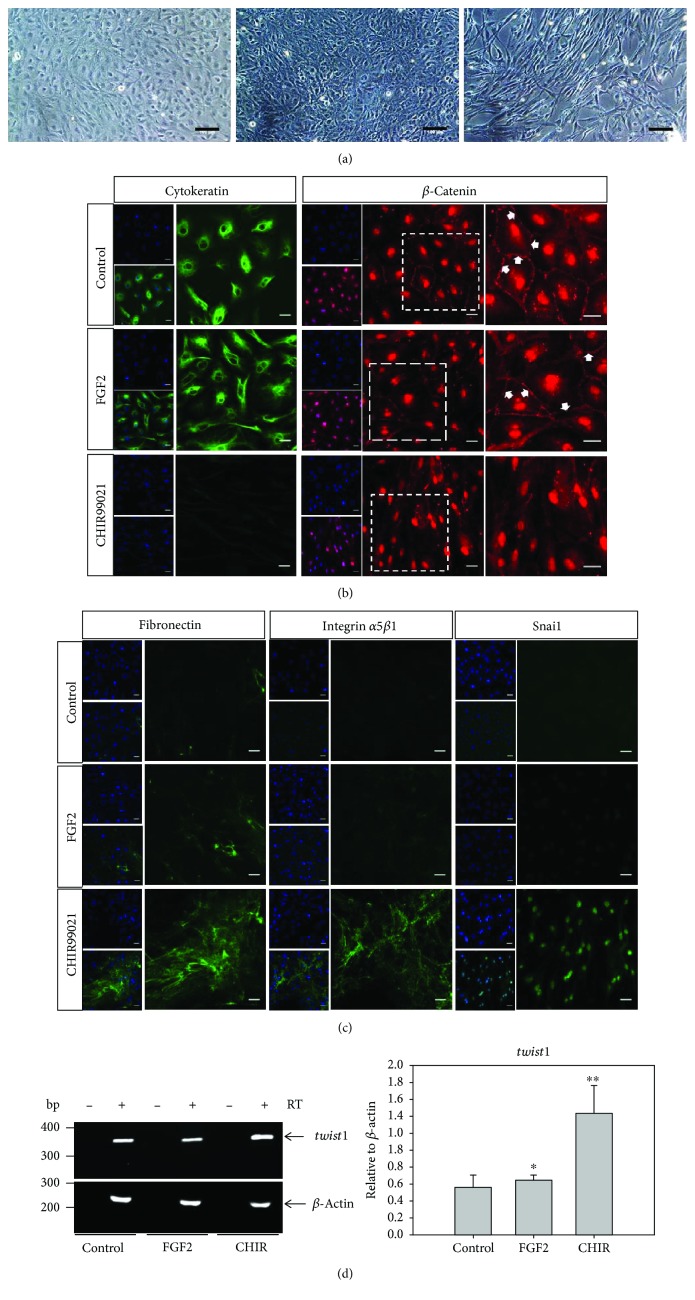
GSK-3 inhibitor (CHIR99021) stimulates EMT in XtiSC cell culture. XtiSCs were cultured in a growth medium supplemented with 25 ng/ml FGF2 or 3 *μ*M CHIR99021 or 0.1% DMSO as a control. (a) After three-day treatment, the morphological change of XtiSCs from cobblestone shape to a long-rod shape in a medium with CHIR99021 was observed. Scale bar: 100 *μ*m. (b, c) Immunofluorescent staining and RT-PCR analysis showed the downregulation of epithelial markers (cytokeratin, *β*-catenin at the plasma cell membrane) (b) and the increase of mesenchymal markers (fibronectin, integrin *α*5*β*1, Snai1, and *twist1*) (c, d). Arrows show the expression of *β*-catenin at the plasma cell membrane. Nuclei stained with DAPI. Scale bar: 20 *μ*m. Results are representative of three biological replicates; ^∗^*p* < 0.005, ^∗∗^*p* < 0.001.

**Figure 2:**
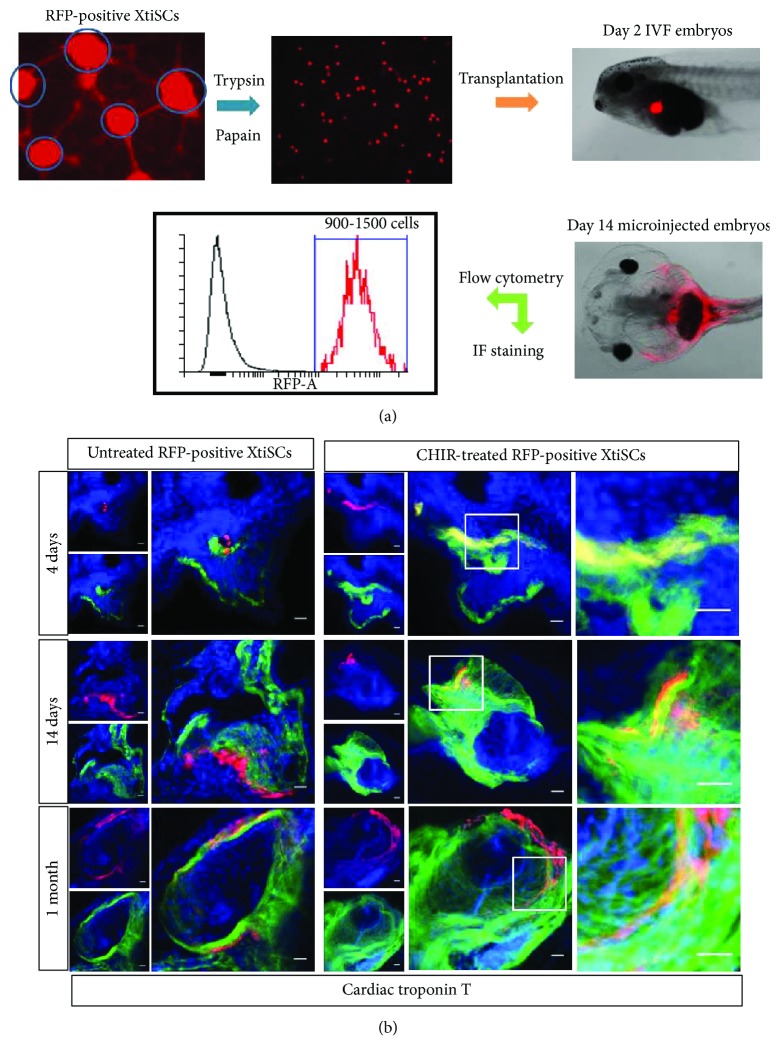
*In vivo* differentiation of EMT-induced XtiSCs into cardiomyocytes. (a) Experimental scheme. RFP-positive XtiSCs were cultured in a growth medium supplemented with 3 *μ*M CHIR99021 or 0.1% DMSO as a control for 3-4 days before transplantation into 2-day-old tadpoles. (b) At the 4th, 14th, or 30th day postinjection (dpi), tadpoles were fixed and sectioned for double staining with antibodies against red fluorescent protein and cardiac troponin T labeling cardiomyocytes in the heart. Scale bar: 20 *μ*m. Nuclei stained with DAPI. Results are representative of four biological replicates.
