# Supplementary material for: Epithelial-Mesenchymal Transition Promotes the Differentiation Potential of Xenopus tropicalis Immature Sertoli Cells
Source: Stem Cells Int. 2019 May 5;2019:8387478. doi: 10.1155/2019/8387478 (PMC6525813; doi:10.1155/2019/8387478)
Supplement: Supplementary Materials — Supplementary Figure 1: phase-contrast images of XtiSCs on microscopic glass coated with various materials. CHIR99021-treated XtiSCs were able to grow only on the collagen type I-coated glass but not on the noncoated or poly-L-lysine-coated glass. In contrary, cells with 0.1% DMSO (control) were not affected by the coating materials. Scale bar: 100 μm. Supplementary Figure 2: (A) immunofluorescent images of mesenchymal interfilament (vimentin) in XtiSCs untreated or treated with CHIR99021 or FGF2. (B) Flow cytometry analysis of surface marker CD29, typical for the mesenchymal cells. GSK-3 inhibitor has no effect on the expression of both proteins. Scale bar: 20 μm. Supplementary Figure 3: XtiSCs became senescent after 3 days of treatment with TGF-β1. Phase-contrast images (A), X-gal (B), and phalloidin (F-actin) staining (C) showed the XtiSC change of shape into bigger and flat cells and the increase of galactosidase activity, a senescent marker after TGF-β1 treatment. XtiSCs untreated or treated with TGF-β1 still expressed the epithelial intermediate filament, cytokeratin (D). Phase-contrast images with scale bar: 100 μm. Nuclei were stained with DAPI. Immunofluorescent images with scale bar: 20 μm. Supplementary Figure 4: adipocyte differentiation of XtiSCs as evidenced by oil red staining after 14 days in induction medium. Positive cells containing red oil droplets appeared in all the cell groups. However, the efficiency of adipocyte induction was quite low. Scale bar: 100 μm. Supplementary Figure 5: immunofluorescent images of cross-sections of injected tadpoles with CHIR-treated cells. Double staining of antibodies against the neuronal surface marker or tropomyosin (a marker of muscle cells) (mouse, green) and RFP (rabbit, red) was employed on the vibratome sections of transplanted tadpoles after 15 days. Scale bar: 50 μm. DAPI (blue) was used for staining cell nuclei. Supplementary Table 1: primer sequences for RT-PCR of Xenopus tropicalis transcripts. Supplem [file 8387478.f1.pdf]

Supplementary figure 1

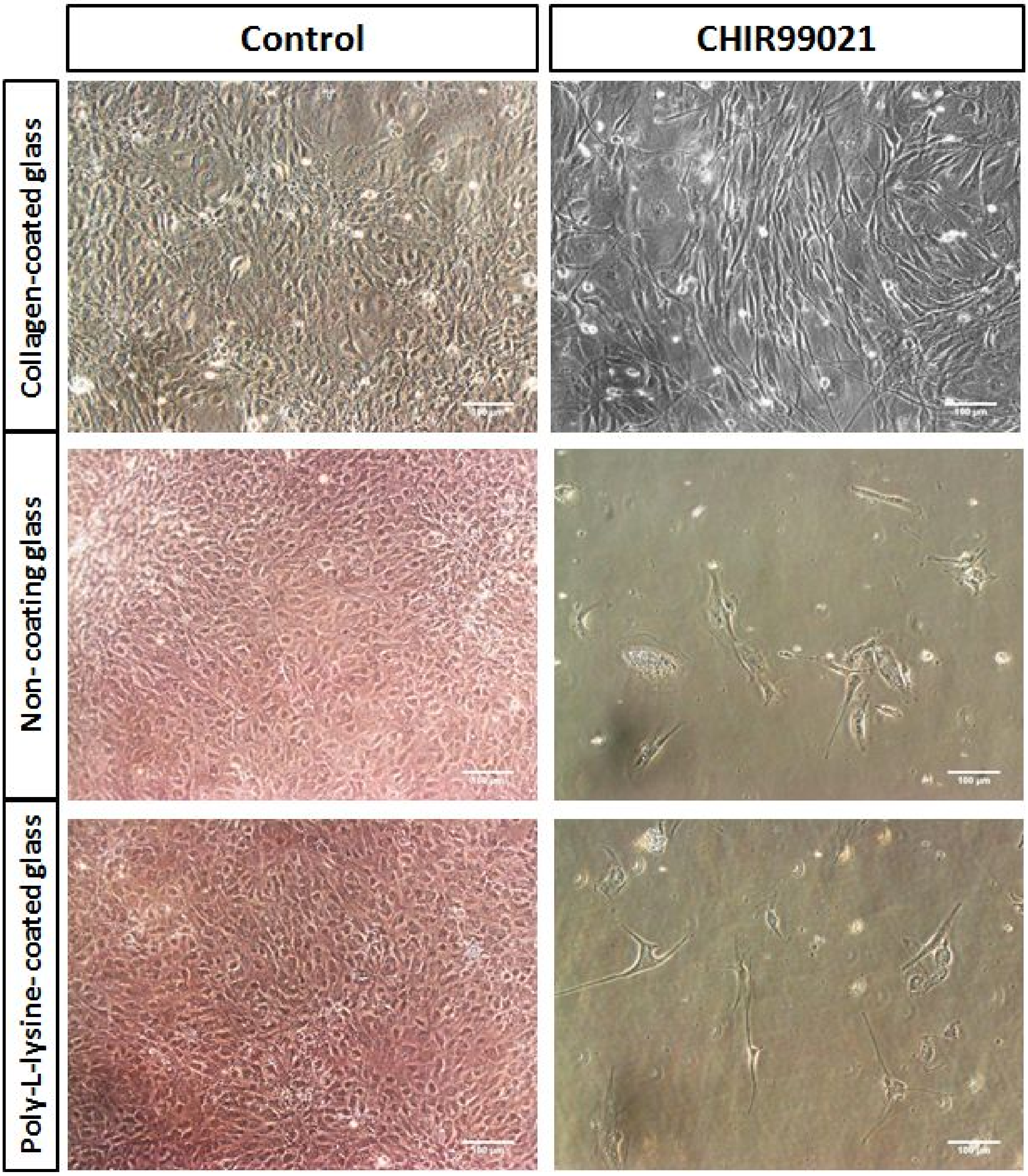

Supplementary figure 1: Phase-contrast images of XtiSCs on microscopic glass coated with various materials. CHIR99021- treated XtiSCs were able to grow only on the collagen type I-coated glass, but not on the non coated or poly-L-lysine coated glass. In contrary, cells with 0.1% DMSO (control) were not affected by the coating materials. Scale bar: 100 µm.

Supplementary figure 2

A.

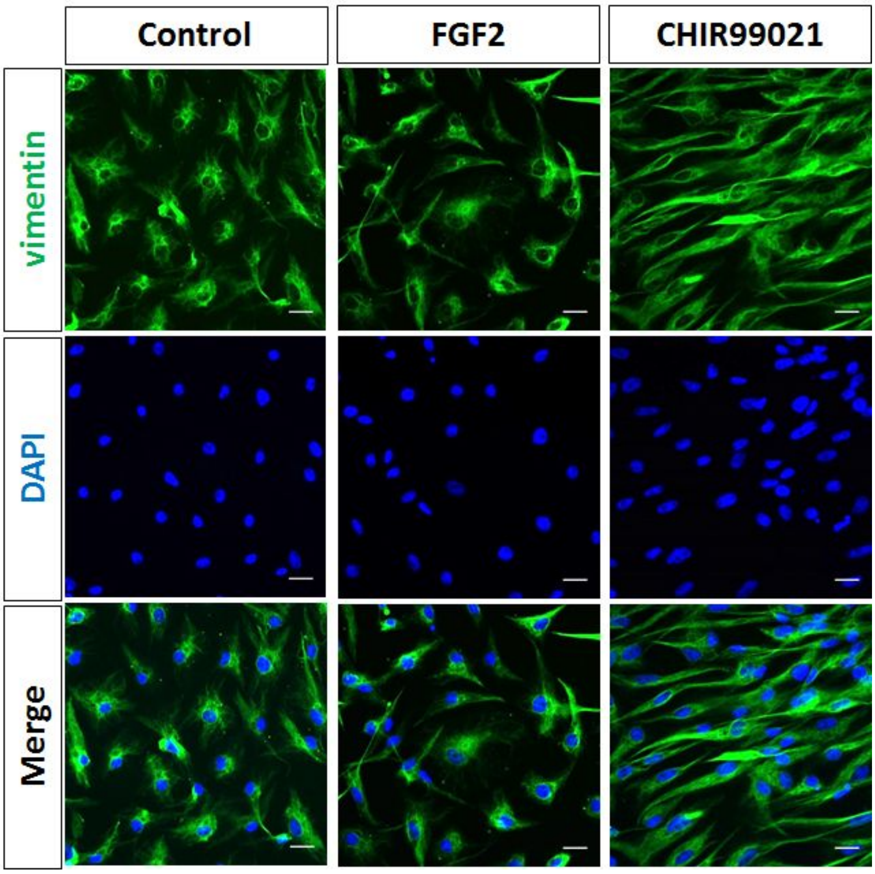

B.

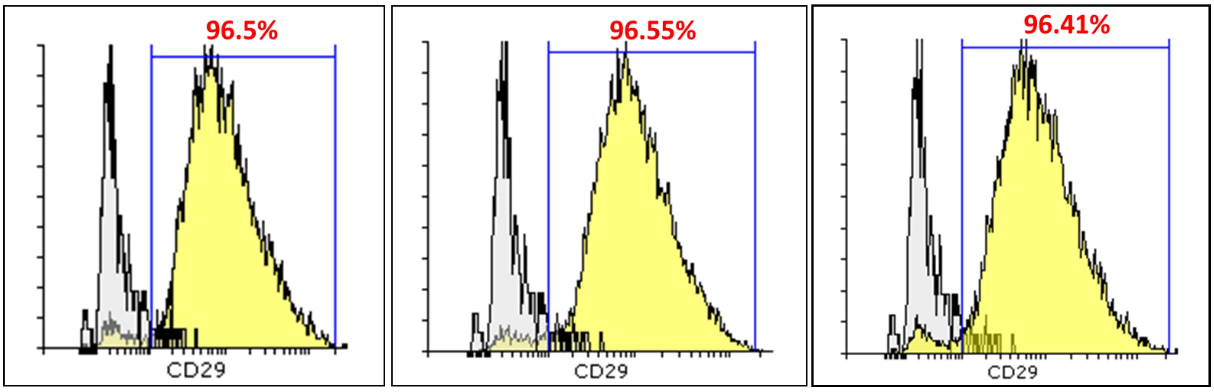

Supplementary figure 2: (A) Immunofluorescent images of mesenchymal interfilament (vimentin) in XtiSCs untreated or treated with CHIR99021 or FGF2. (B) Flow cytometry analysis of surface marker CD29, typical for the mesenchymal cells. GSK3 inhibitor has no effect on the expression of both proteins. Scale bar: 20 µm.

Supplementary figure 3

A.

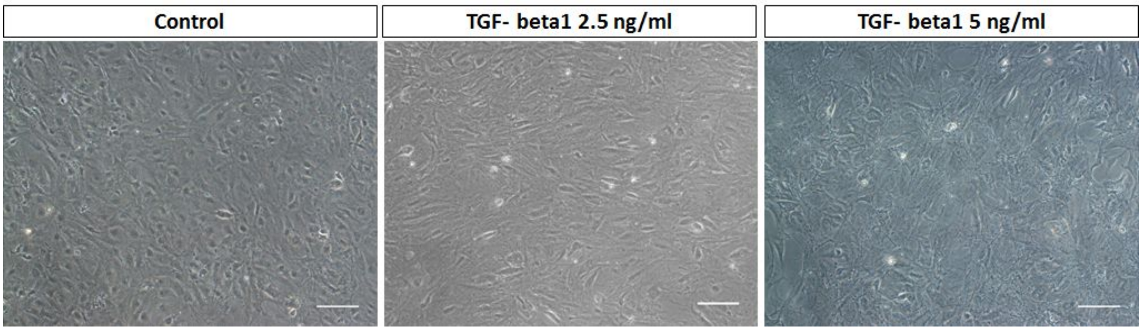

B.

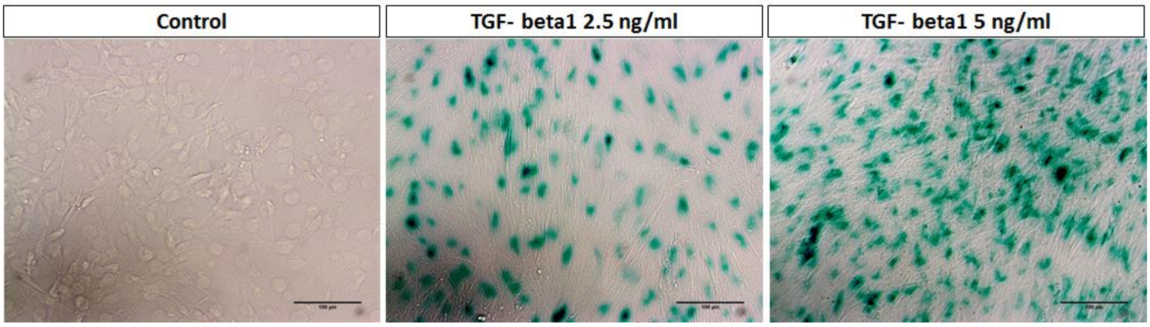

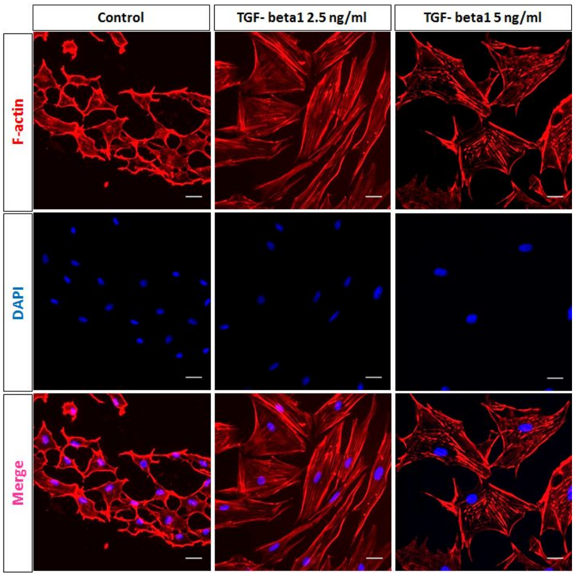

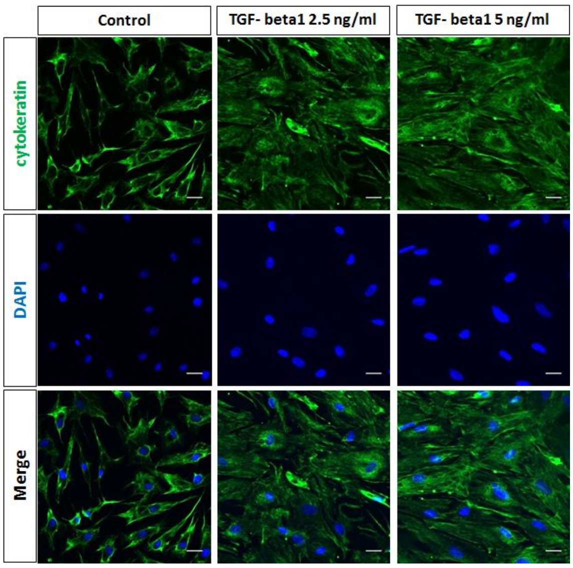

C. **D.**

Supplementary figure 3: XtiSCs became senescent after 3 days of treatment with TGF-β1. Phase-contrast images (A), X-gal (B) and Phalloidin (F-actin) staining (C) showed the XtiSCs change of shape into bigger and flat cells, and the increase of galactosidase activity, a senescent marker after TGF-β1 treatment. XtiSCs untreated or treated with TGF-β1 still expressed the epithelial intermediate filament, cytokeratin (D). Phase-contrast images with scale bar: 100 µm. Nuclei were stained with DAPI. Immunofluorescent images with scale bar: 20 µm.

Supplementary figure 4

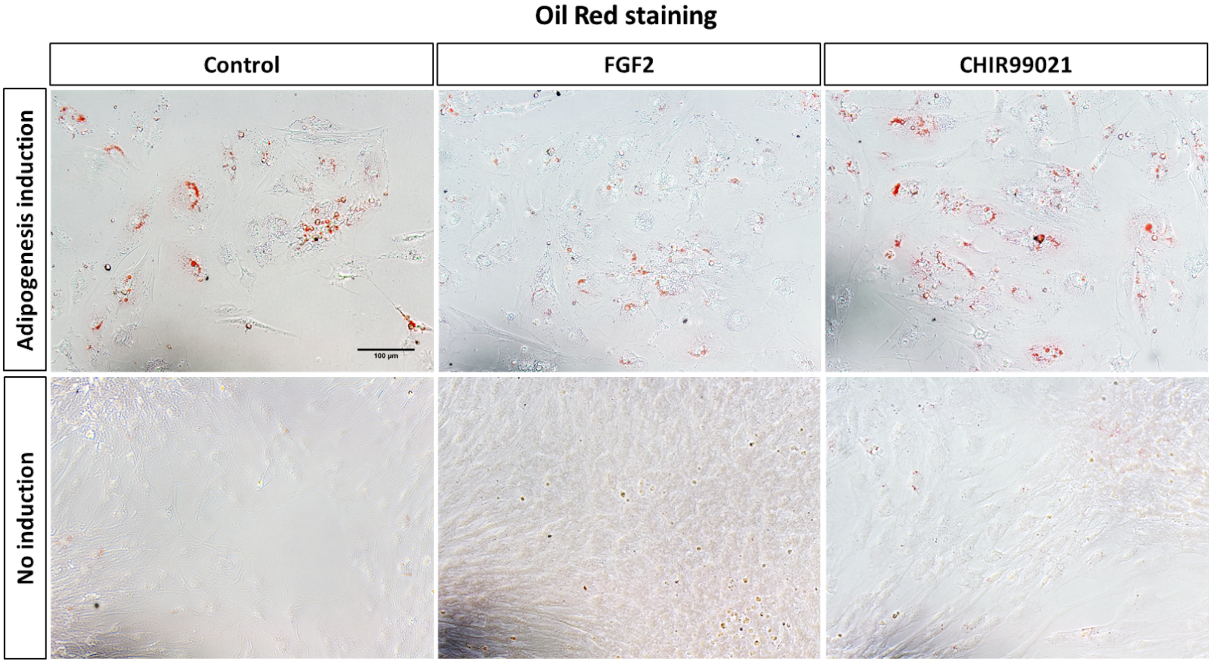

Supplementary figure 4: Adipocyte differentiation of XtiSCs as evidenced with oil red staining after 14 days in induction medium. Positive cells containing red oil droplets appeared in all the cell groups. However, the efficiency of adipocyte induction was quite low. Scale bar: 100 µm.

Supplementary figure 5

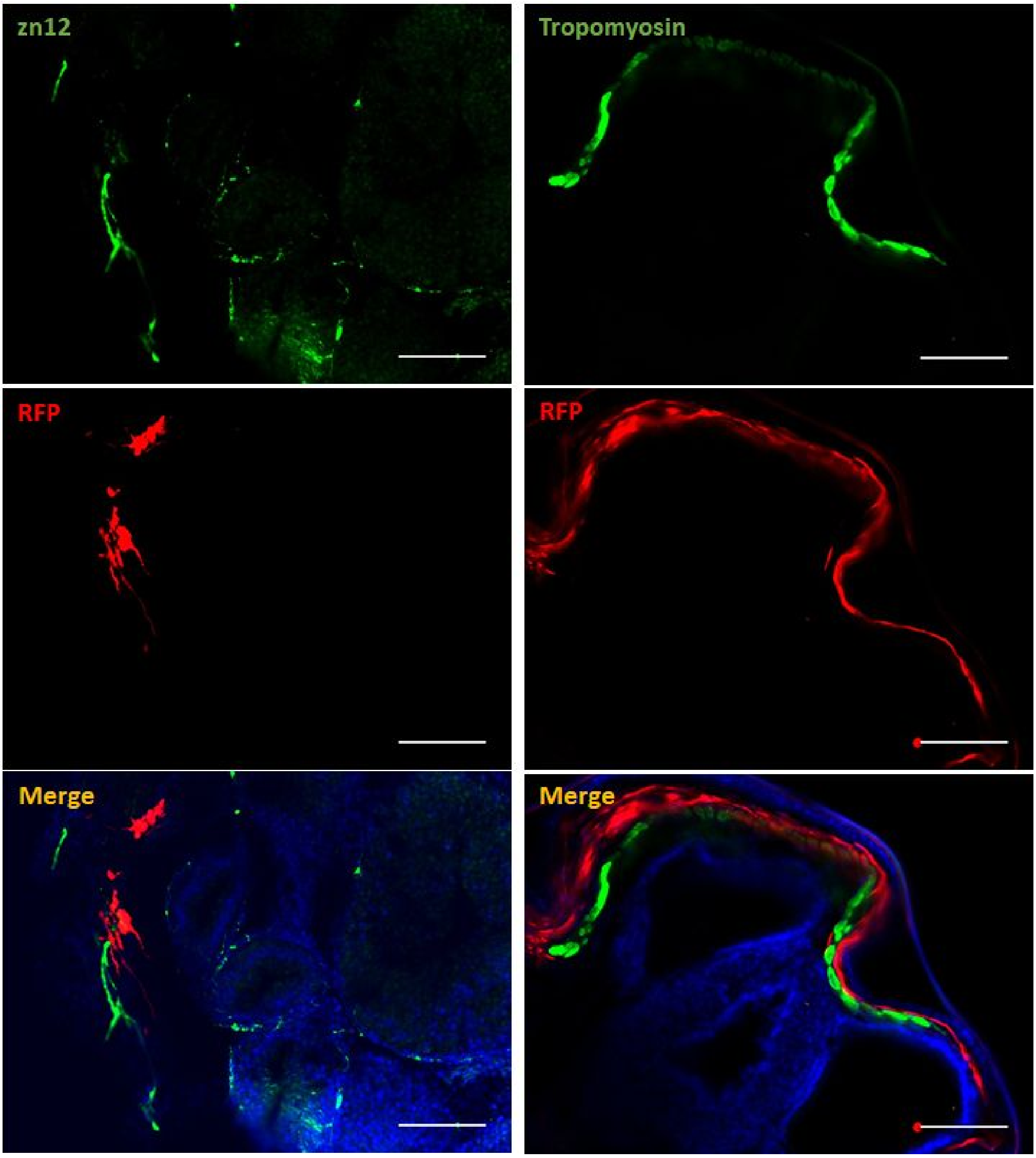

Supplementary figure 5: Immunofluorescent images of cross-sections of injected tadpoles with CHIR-treated cells. Double staining of antibodies against neuronal surface marker or tropomyosin (a marker of muscle cells) (mouse, green) and RFP (rabbit, red) were employed on the vibratome sections of transplanted tadpoles after 15 days. Scale bar: 50 µm. DAPI (blue) was used for staining cell nuclei.

Supplementary table 1

Primer sequences for RT-PCR of *Xenopus tropicalis* transcripts

Gene symbol	Gene name	Gene transcript	Primer F<br>Primer R	Size (bp)
cd44	CD44 molecule	ENSXETT00000016456	CCC TGG GCA ATA ACG ATT CC<br>ATC GGT GAC CTC TCC TGG AT	572
twist1	Twist-family bHLH transcription factor 1	ENSXETT00000054522	TCTCCCCAGTAGACAGTCTAA<br>AGACCTGGCAGAGAAAGTCG	351
actb	Actin, beta	ENSXETT00000006761	CCAAGCTGTGTTGTCCCTGT<br>GCTGTGGTGGTGAAGCTGTA	235

Supplementary table 2: Antibodies used in immunofluorescent staining

Antigen	Species	Provider	Cat./Clone No.	Reference/ Certificate No.	Dilution
Cytokeratin	Mouse	Developmental Studies Hybridoma Bank	1h5	Klymkowsky et al,. 1987	2 µg/ml
Fibronectin	Mouse	Developmental Studies Hybridoma Bank	MT4	Jackie D. Nace and Roy A. Tassava, 1995	2 µg/ml
Beta-catenin	Rabbit	Sigma-Aldrich	C2206		1:500
Integrin alpha 5 beta 1 (fibronectin receptor)	Mouse	Developmental Studies Hybridoma Bank	P8D4	9/26/16	2 µg/ml
Integrin beta 1 (CD29)	Mouse	Developmental Studies Hybridoma Bank	8C8	3/14/13	2 µg/ml
Snai1	Mouse	Santa Cruz Biotechnology	sc271977	E2716	1:100
Snai1	Mouse	St John's Laboratory	STJ95716		2 µg/ml
Zeb1	Mouse	Novus Biologicals	2A8A6	NBP2-23484SS A-1	1:100
Sox2	Mouse	Santa Cruz	sc-365823	E2716	1:100
Cardiac troponin T	Mouse	Developmental Studies Hybridoma Bank	CT3	Dagle et al., 2003	2 µg/ml
STAT3	Mouse	Developmental Studies Hybridoma Bank	PCRP-STAT3-2F12	11/5/15	2 µg/ml
Vimentin	Mouse	Developmental Studies Hybridoma Bank	14h7	Joseph A. Dent, 1989.	1 µg/ml
alpha-smooth muscle actin	Mouse	Sigma-Aldrich	A2547	Validate by staining in testis section	1:400
Phalloidin (F-actin)		Thermo Fisher Scientific		1668974	1:100
Sox9	Rabbit	Sigma-Aldrich	HPA001758	Anwar El Jamil, 2008	1:300
Red fluorescence protein	Rabbit	Evrogen	AB233	23301291014	1:5000
Red fluorescence protein	Mouse	Thermo Fisher Scientific	MA5-15257	QB205317	1:500
Mouse IgG-Alexa Fluor®	Goat	Thermo Fisher Scientific	A11001	1664729	1:500

488 conjugate					
Rabbit IgG-Alexa Fluor® 594 conjugate	Goat	Thermo Fisher Scientific	A11012	1678830	1:500
Mouse IgG-Alexa Fluor® 594 conjugate	Goat	Thermo Fisher Scientific	A11032	419361	1:500
Rabbit IgG-Alexa Fluor®488 conjugate	Goat	Thermo Fisher Scientific	A11034	870976	1:500
